# Microplastic quantification in Sabellaria reefs: a validated protocol for extraction from biogenic agglutinated matrices

**DOI:** 10.1007/s11356-026-37573-7

**Published:** 2026-03-14

**Authors:** Giusto Lo Bue, Rosa Maria Festa, Maya Musa, Maria Pia Riccardi, Alessandro Croce, Agnese Marchini, Nicoletta Mancin

**Affiliations:** 1 Department of Earth and Environmental Sciences, University of Pavia, via Ferrata 1, Pavia, 27100 Italy; 2 SSD Research Laboratories, Research and Innovation Department (DAIRI), Azienda Ospedaliero-Universitaria SS. Antonio e Biagio e Cesare Arrigo, Via Venezia 16, Alessandria, 15121 Italy; 3 Department of Science and Technological Innovation, University of Eastern Piedmont, Viale T. Michel 11, Alessandria, 15121 Italy

**Keywords:** Microplastic, Protocol validation, Agglutinated matrix, Sabellariid reef

## Abstract

**Abstract:**

Microplastic pollution affects all marine ecosystems, particularly coastal areas inhabited by sedentary reef-building organisms that rely on sand grains to build arenaceous reefs (e.g., Sabellariid polychaetes). These agglutinated reefs passively trap microplastics, thus increasing the potential risk to benthic organisms that live on and within the reef. An accurate quantitative assessment of microplastics accumulated within these arenaceous reefs is currently hindered by a lack of standardized methodologies. This study addresses this gap by developing and validating a reliable and reproducible protocol specifically designed to extract and quantify microplastics cemented within bioconstructed agglutinated matrices. The proposed protocol evaluated digestion procedures aimed at the release of microplastics from agglutinated matrices. The subsequent density extraction procedure was validated via a spiking experiment using both bioconstruction and sediment samples spiked with known quantities of polyethylene terephthalate, polypropylene, and polyvinyl chloride. Scanning electron microscopy and µ-Raman spectroscopy confirmed that the adopted digestion procedures did not alter the plastic polymers. Results also showed that the NaI solution yielded a significantly higher microplastic recovery than NaCl. Notably, microplastic recovery using NaCl was influenced by the initial sample weight, suggesting possible matrix interference at higher sample weights. Our multistep approach provides a validated, cost-effective, and reproducible protocol that improves microplastic quantification in agglutinated matrices. By employing common laboratory equipment and specific procedures, this methodology represents a significant step towards standardizing microplastic pollution monitoring in coastal bioengineered habitats.

**Highlights:**

• A step-by-step protocol for MP density extraction from biogenic agglutinated matrices was validated.

• Agglutinated matrices require disaggregation to release MP, ensuring a correct density extraction.

• Preliminary drying and disaggregation procedures do not alter the chemical integrity of MP.

• NaI solution is significantly more efficient than NaCl for MP extraction.

• Substrate type (sediment vs Sabellariid bioconstruction) had no influence on  density extraction efficiency.

**Supplementary Information:**

The online version contains supplementary material available at 10.1007/s11356-026-37573-7.

## Introduction

For over six decades, plastic has brought countless benefits to industry, economy, trade, sciences, medicine, and daily life. Its high resistance to degradation (Mathalon & Hill 2014) is what makes it so valuable; however, the combination with the proliferation of single-use products has resulted in an exponential growth of plastic waste. Plastic pollution is an economic, environmental, and social concern for present and future generations (Galloway & Lewis 2016): a planetary threat (Villarrubia-Gómez et al. 2018) that requires concerted actions at all levels of government and across multiple geographical scales (Carlini & Kleine 2018).

Among plastic waste, microplastics (MPs) are particularly dangerous given their worldwide distribution. They can reach the environment through direct release (primary MP) and through the degradation of larger plastic objects (secondary MP) (Barnes et al. 2009; O’Brine & Thompson 2010; Browne et al. 2011; Andrady & Koongolla 2022; Andrady et al. 2022). Conventionally, MPs are defined as particles ranging in size from 5 mm down to 1 µm (Frias & Nash 2019). Due to their small size, MPs easily interact with biota hence threatening biodiversity and ecosystem integrity and causing serious socio-economic damages that are often difficult to quantify (Wright et al. 2013; Chaudhry & Sachdeva 2021; Ghosh et al. 2023). Furthermore, MPs could adsorb, convey, and leach chemical compounds (additives such as flame retardants, plasticizers, colorants, heavy metals, or other biocides), microorganisms, and pathogens, increasing the potential toxic effect (Liu et al. 2019; Godoy et al. 2020; Bridson et al. 2023).

The coastal environment is the main entry point for MPs, with subsequent accumulation and temporary storage occurring predominantly in sea-floor sediments (Van Cauwenberghe et al. 2015; Yao et al. 2019; Darabi et al. 2021; Yang et al. 2024). Coastal ecosystems house high biodiversity and perform crucial functions for environmental integrity and human well-being (Liquete et al. 2016; Basconi et al. 2023). Unfortunately, they are also subject to high anthropogenic pressure: coastal urbanization, extensive cultivated areas, fishing, aquaculture, and tourism all contribute to a growing supply of MPs (Muñiz & Rahman 2025). The accurate quantification of MP abundance in marine matrices is crucial for assessing risks and developing effective management strategies (Miller et al. 2017; Lusher et al. 2020; Bäuerlein et al. 2022; Lo Bue et al. 2023), yet significant methodological discrepancies still persist: the quantification of MP abundance can be over- or underestimated, and collected data are often very difficult to compare due to the lack of standardized procedures in sample preparation and MP extraction, detection, and quantification (Zhao et al. 2025).

Despite the existence of guidelines for marine sediment sampling (Cheshire et al. 2009; Galgani et al. 2013; Lippiatt et al. 2016; MSFD Technical Group on Marine Litter, 2023), the subsequent procedures for sample preparation and analysis are not standard. Each step, from sample drying (Munno et al. 2017; Al-Azzawi et al. 2020) to organic matter removal and MP extraction (Prata et al. 2019; Pfeiffer and Fischer 2020), can introduce uncertainties.

In particular, the MP extraction process is not unequivocally defined. Several extraction techniques have been applied: density separation by hypersaline solutions (Hanvey et al. 2017) or by oil (Crichton et al. 2017), elutriation (Claessens et al. 2013), and electrostatic extraction (Felsing et al. 2018), all of which produce data that are difficult to compare. Density separation using a hypersaline solution is the most common technique, but its efficiency depends on the salt used. Sodium chloride (NaCl), suggested in several guidelines because it is less expensive and non-toxic, fails to recover denser plastic polymers, while high-density brine solutions, such as sodium iodide (NaI) or zinc chloride (ZnCl₂), are more efficient but also more expensive and toxic (Hidalgo-Ruz et al. 2012; Frias et al. 2018; MSFD Technical Group on Marin Litter, 2023).

The type of environmental matrix being investigated clearly influences the choice of density separation techniques. Agglutinated matrices formed by reef-building organisms have the peculiarity of containing MPs that are cemented within the sand grains forming the bioconstruction. Their extraction, therefore, involves the preliminary disaggregation of the bioconstruction to release MPs. Very few studies have explored this topic (da Costa et al. 2021; Lo Bue et al. 2023; Schuab et al. 2023; Barra et al. 2025; Santos et al. 2025), although the same authors have documented how bioengineered habitats could represent MP sinks with consequences that are still unknown (Williams et al. 2022; Lo Bue et al. 2025).

The honeycomb worm and ross worm, *Sabellaria alveolata* (Linneus, 1767) and *Sabellaria spinulosa* (Leuckart, 1849), are ecosystem engineer species that form arenaceous reefs in the coastal environment of temperate seas by cementing sandy grains. Sabellariid bioconstructions are a model system in the littoral environment that can work as a permanent MP trap thanks to the specific building behavior of the polychaetes. These sedentary organisms actively construct their arenaceous tubes by selecting grains based on their size, shape, density, and buoyancy (Lo Bue et al., 2022; Lisco et al., 2023). Lo Bue et al. (2023, 2025) demonstrated that sabellariid reefs passively trap MPs without any direct action by the polychaetes. Unfortunately, this passive entrapment makes the reef a physical sink for anthropogenic contaminants. Understanding this non-selective mechanism is a critical issue, as *Sabellaria* reefs are widely distributed along the temperate coasts of both Atlantic and Mediterranean basins where they sustain biodiversity (Dubois et al. 2002, 2006; Ventura et al. 2024) and protect coasts from erosion (Lisco et al. 2017, 2020). Thus, they have been formally defined as a “valuable marine habitat” under Annex I of the EU Habitats Directive (92/43/EEC) as “Reefs” (Habitat code 1170) (Gubbay et al. 2016). Despite the ecological role they play, a dedicated protocol for studying MP pollution in sabellariid ecosystems is currently lacking. To address this gap, our work aims at providing a validated, reliable, and reproducible protocol specifically designed to extract and quantify MPs from agglutinated matrices.

The validation of the proposed protocol is carried out in two steps: the first step investigates the potential MP alteration induced by the procedures applied to disaggregate the agglutinated matrix and release MPs; the second step, performed through a laboratory experiment, tests MP extraction efficiency by using (i) low-density (NaCl) *versus* high-density (NaI) salt solutions, (ii) bioconstruction samples vs sea-floor sediments, and (iii) different sample quantities (large vs small sample). This last point specifically aims to address the need to work with small samples since biogenic reefs are valuable habitats and the collection of large samples should be discouraged.

## Materials and methods

### Sample preparation, MP production, and polymer characterization

Bioconstruction and sediment samples used in the validation experiment were collected from the largest reef built by *Sabellaria spinulosa* (Leuckart, 1849) in the Mediterranean Sea: the Torre Mileto reef, recently proposed as a “Site of Community Importance” (Lisco et al. 2021) to be protected by the Habitat Directive (92/43/EEC).

To minimize environmental disturbance to the bioconstruction, 20 small samples (~ 250 mL) were collected from an area of ~ 200 m^2^ using a stainless steel spatula; a small corer was used for sediment samples.

Once in the laboratory, all samples were dried at 40 °C for at least 36 h until a constant weight was reached. The constant weight was operationally defined as the condition where two consecutive weight measurements, taken 6 h apart, showed no substantial difference, ensuring complete evaporation. Then, dried samples were digested in 30% hydrogen peroxide (H_2_O_2_ 130 Vol) for 48 h to remove both the organic cement (disaggregating the bioconstruction tubes) and the organic matter present in sediment samples. The volume of peroxide used was twice the volume of each treated sample, an amount that preliminary observations showed to be sufficient for achieving complete digestion of the organic material in the investigated matrices. After digestion, any MP originally present in the matrices was removed through density separation by using a NaI brine solution (Nuelle et al. 2014; Kedzierski et al. 2019). The resulting “cleaned” sand residues were dried again, weighed, and stored for subsequent use in the spiking validation experiment.

Three different types of MPs were artificially produced in the laboratory by crumbling common consumer plastic products: polypropylene (PP), polyvinyl chloride (PVC), and polyethylene terephthalate (PET). These are among the most employed polymers in the manufacturing industry, and consequently, they are the most common plastics dispersed and found in marine environments (Geyer et al. 2017). The polymers also exhibit heterogeneous densities (PP, 0.855–0.946 g cm^−3^; PVC, 1.1–1.35 g cm^−3^; PET, 1.38 g cm^−3^—Morét-Ferguson et al. 2010) which makes them ideal for validating the extraction efficiency of density-based separation. The plastic items were also differently colored (PP, light blue; PET, dark blue; and PVC, orange), allowing for straightforward visual identification during subsequent optical microscopy analysis for census counts.

The MP production method was designed to address the specific plastic polymers’ glass transition temperatures (Wilkes et al. 2005). Brittle fragments of PP and PVC were obtained by freezing larger items in a common household freezer (−20 °C), then crumbling them in a metal coffee grinder. PET particles were instead plunged into a liquid nitrogen bath (−196 °C) for 10 min and subsequently cryomilled using a Fritsch Pulverisette 11 mill (10 cycles of speed of 10,000 rpm for 30 s). The resulting MPs were then sorted using two overlapped steel sieves to retain the size fraction > 0.250 mm and < 1.41 mm. This size fraction was chosen because it is known that *Sabellaria* polychaetes mainly use sand grains of this size to build the arenaceous tubes of the bioconstruction.

After sieving, MPs were placed in an oven at 40 °C for at least 36 h and subsequently treated with hydrogen peroxide for 48 h to simulate the digestion process and thus evaluate possible polymer changes induced by the preliminary procedures adopted to prepare samples.

The chemical structure of plastic polymers was investigated at each preparation phase by using a HORIBA Jobin Yvon µ-Raman spectrometer which provided a vibrational “fingerprint” for each polymer. Subsequently, the surface morphology of the same MP was examined with a Tescan Mira 3XMU Scanning Electron Microscope (SEM).

µ-Raman analyses were carried out on isolated MPs that were placed directly on aluminum foil. The instrument was equipped with 532 nm (green) and 785 (near-Infrared) laser sources and two gratings, 600 l mm^−2^ and 1800 l mm^−2^. Specifically, PP fragments were analyzed using the 1800 l mm^−2^ grating, with 10 s of integration time for 1 cycle; while for PET and PVC, the 600 l mm^−2^ grating was used with 5 s of integration time for 5 cycles, due to the different lasers used on the different polymers (indeed, the optimisation of the Raman acquisition set-up was considered part of the protocol development). In order to evaluate possible alterations in the polymeric structures correlated with the extraction protocol’s steps, several MPs were analyzed by both SEM and µ-Raman spectroscopy at the different stages of extraction: untreated MPs (after sieving), after 40 °C temperature treatment, and after hydrogen peroxide digestion (40 °C + H_2_O_2_).

### Experimental design

**Fig. 1:**
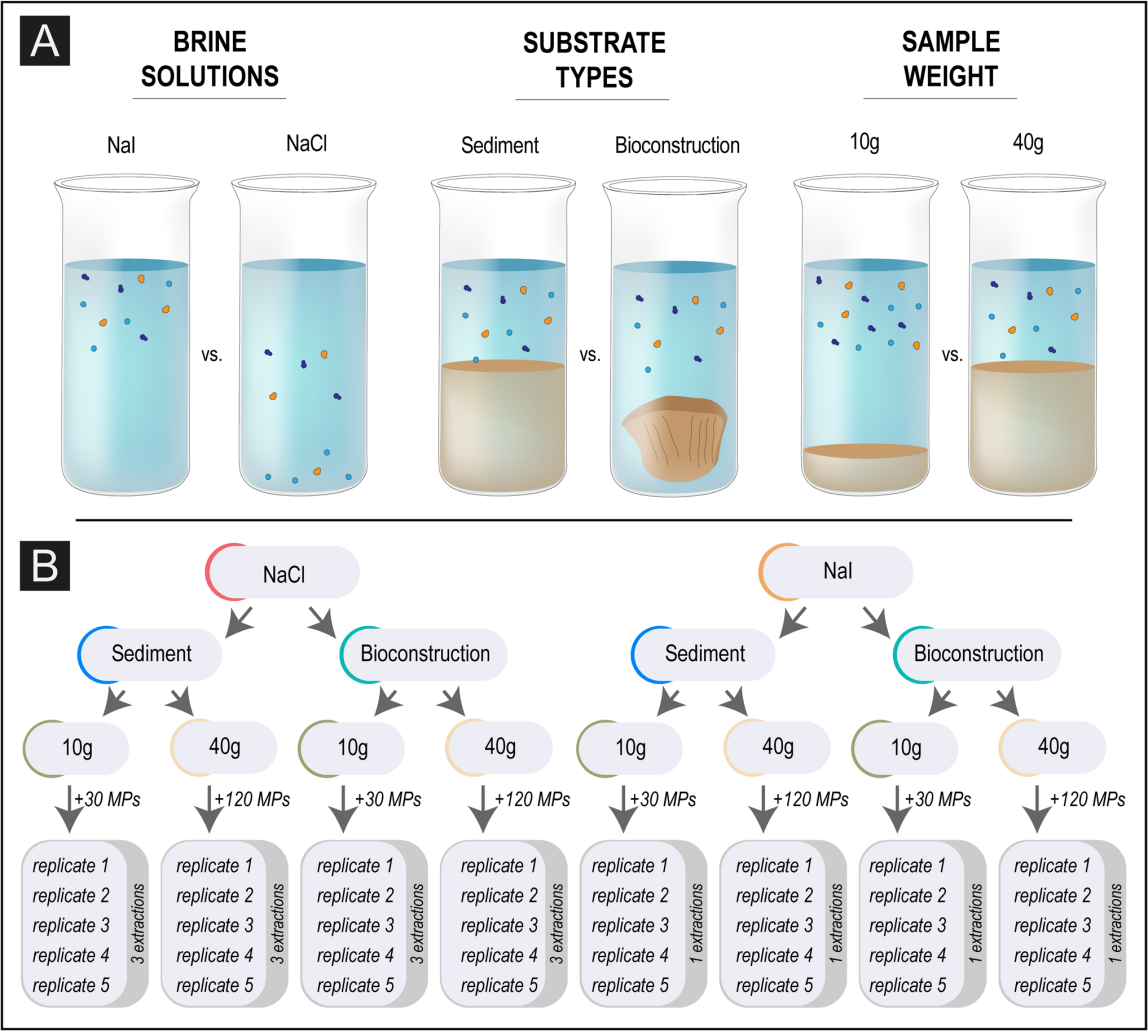
Synthesis of the adopted experimental design. **A** Experimental conditions to be tested (2 types of salt solution, 2 types of substrate, and 2 quantities of substrate). **B** Structure and number of replicates of the spiking experiment

The experimental plan (Fig. 1A, B) was designed using 40 replicates to provide eight different treatment combinations by testing two substrates (bioconstruction and sediment) and two sample weights (10 g and 40 g), with each combination being tested against both NaCl and NaI solutions.

The spiking was performed under a laminar flow hood, paying close attention to avoid the loss of introduced MPs. Thirty MPs (10 of each polymer type) were added to 10 g of each substrate (bioconstruction and sediment); a higher number of MPs (120 in total, 40 of each polymer type) were added to larger samples consisting of 40 g of each substrate. A total of 3200 MPs were used. MPs were intentionally added to sediment and bioconstruction samples in known quantities of size (from 0.250 to 1.41 mm), shape (fragments), and color (PP, light blue; PET, dark blue; and PVC, orange) to facilitate visual recognition and census count under the microscope. Therefore, procedural blanks were not used as MP recovery rates were tested by exclusively quantifying the polymers (PET, PP, and PVC fragments) that were added to the samples and that were clearly different from possible laboratory contaminants such as airborne fibers.

The low-density NaCl solution was obtained by dissolving 71.8 g of salt in 200 mL of hot tap water (~ 80 °C), resulting in an average density of 1.18 ± 0.0832 g cm^−3^ (Besley et al. 2017; Menendez et al. 2022; Lo Bue et al. 2023). The high-density NaI solution was prepared by dissolving 300 g of salt in 200 mL of hot water, achieving a much higher average density of 1.70 ± 0.0566 g cm^−3^ (Van Cauwenberghe et al. 2013; Kedzierski et al. 2017). To prevent contamination from MPs potentially present in the salts, both solutions were filtered through a 0.23-µm cellulose ester membrane prior to use (Zhang et al. 2020; Lo Bue et al. 2023).

The spiked samples were suspended in 200 mL of each brine solution contained in glass beakers. The mixture was manually homogenized with a stainless steel spatula for 5 min, sonicated for 30 min in an ultrasonic bath at room temperature (Lo Bue et al. 2023), and finally left to rest for a period of 6 to 12 h under a hood to allow the heavier particles to settle. The beakers were covered with aluminium foil to prevent possible MP loss.

MP extraction was performed by vacuum filtering the upper 100 mL of the supernatant onto cellulose ester filters (pore size 0.23 µm, Millipore). The extraction process was repeated three times for samples processed using the lower-density NaCl solution (Ng & Obbard 2006; Browne et al. 2011; Claessens et al. 2011). By contrast, a single filtration was applied for samples processed with the high-density NaI solution (Claessens et al. 2013; Dekiff et al. 2014; Nuelle et al. 2014; Quinn et al. 2017). The obtained filters were placed in sealed glass Petri dishes to prevent any loss of the extracted MPs.

### Data collection and statistical analysis

For MP counting, each filter was examined under a stereomicroscope (Leica M60) at 40× magnification to quantify both relative abundance (calculated as the percentage of the total MPs added to samples) and absolute abundance (defined as the number of MPs counted on the filter relative to the initial sample weight). After quantifying polymer types based on color, all MPs were also individually measured (largest linear dimension of each fragment) using an ocular integrated micrometer scale.

For the subsequent univariate analysis, the relative MP abundances were used to calculate the recovery efficiency of density separation expressed as %. In detail, to evaluate how different experimental conditions influenced the effectiveness of MP extraction, a three-way analysis of variance (ANOVA) was employed after verifying assumptions (normality and homoscedasticity). Specifically, the ANOVA’s aim was to determine if statistically significant differences existed in the mean recovery efficiency due to the main effects of three distinct factors (Fig. 1): salt type, substrate type, and sample weight. The null hypothesis for each main effect was that the mean recovery efficiency was equal across the levels of that factor. Furthermore, interaction effects were tested to observe whether the impact of one factor was consistent across the levels of the others. Consequently, the analysis also tested the null hypotheses associated with the two-way interactions (salt:substrate, salt:weight, and substrate:weight) and the three-way interaction (salt:substrate:weight).

For multivariate analysis, we utilized MP absolute abundance (items g^−1^ dry weight). These abundances were then classified according to polymer type (PET, PP, PVC) and particle size (0.25–0.5 mm; 0.5–1 mm; 1–2 mm, following Blott & Pye, 2012) within each polymer type. Redundancy analysis (RDA) was performed to explore the influence of salt, substrate, and sample weight on MP abundances (items g^−1^ d.w.) for each polymer type and size class. RDA model significance, individual axis significance, and marginal effects of experimental factors were assessed through multiple permutation tests.

To assess the influence of salt type, substrate, and sample weight on the multivariate composition of extracted MPs, permutational multivariate analysis of variance (PERMANOVA) was performed based on Euclidean distances calculated from the multivariate dataset. The adopted approach was analogous to the univariate one, testing the main effects and all interactions between salt type, substrate, and sample weight. Significance was evaluated using 9999 unrestricted permutations. Complementary to the PERMANOVA, the homogeneity of multivariate dispersion among groups was tested using the PERMDISP routine (permutational test of multivariate homogeneity of group dispersions) based on distances to group centroids derived from the same similarity matrix (Anderson 2001; Anderson et al. 2006).

Statistical analyses were carried out using the software R version 4.4.1 (R Core Team, 2024). The R packages employed were “ggplot2” (Wickham 2016) for graphical representations, “tidyr” (Wickham et al. 2019) and “dplyr” (Wickham, H., Franois, R., Henry, L., Müller, K. and Vaughan, D., 2023) for data manipulation, and “vegan” (Oksanen et al. 2001) for univariate and multivariate analyses. When possible, test outputs were double-checked with PRIMER-e + PERMANOVA software (V. 6.0) (Clarke & Gorley, 2006). Statistical significance was set at *p* < 0.05 for all tests.

## Results and discussions

### Evaluation of MP alterations induced by preliminary laboratory procedures

**Fig. 2:**
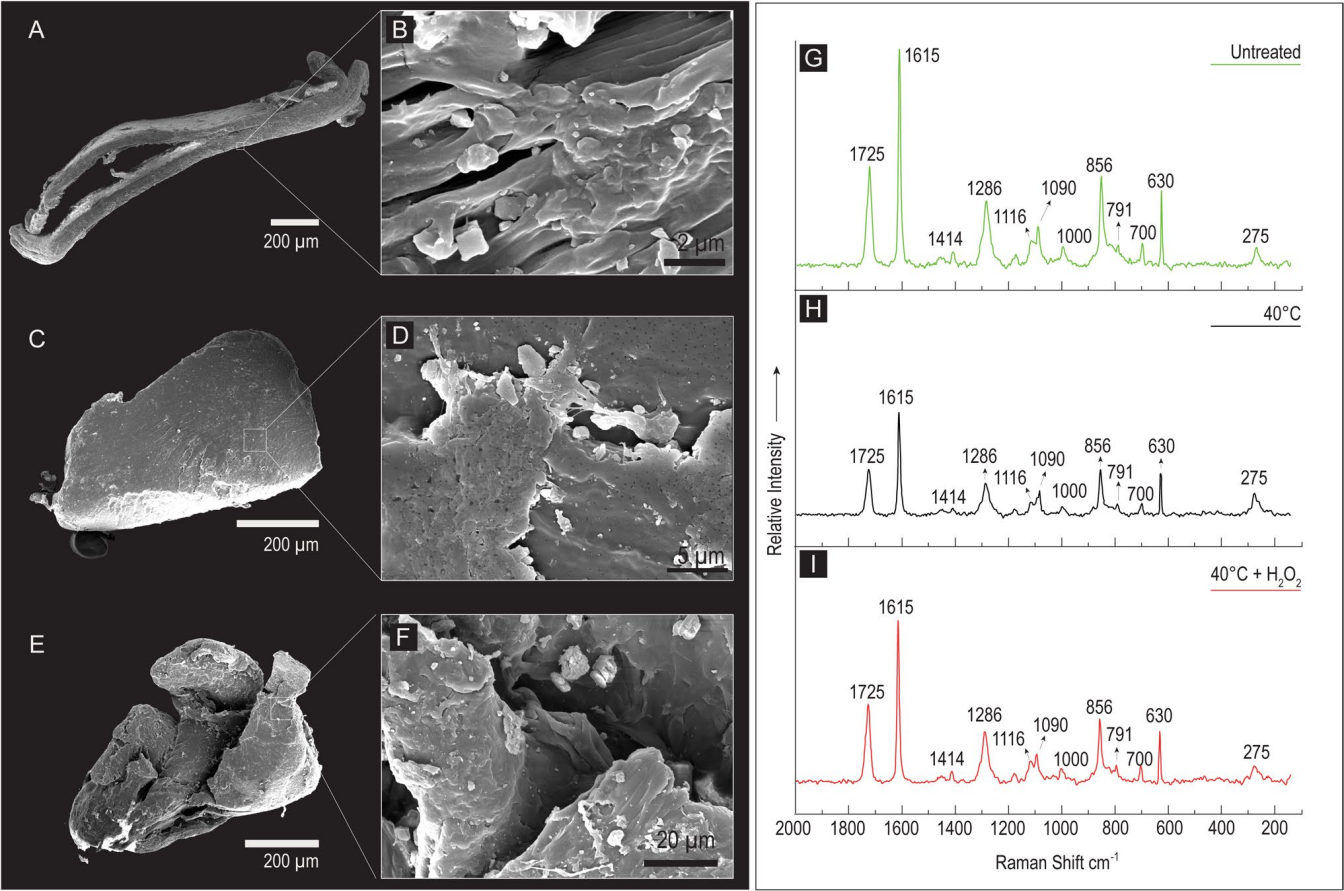
SEM images in secondary electrons and Raman spectra of three polyethylene terephtalate (PET) fragments. **A** An untreated PET fragment with an elongated shape; **B** detail of the fragment surface, characterized by a fibrous texture and several sub-micrometer particles; on the right in green (G), the corresponding Raman spectrum. **C** A regular shaped PET fragment observed after the drying process (48 h at 40 °C); **D** detail of the surface showing cracks; on the right in black (H), the corresponding Raman spectrum. **E** An irregular shaped PET fragment observed after the drying and digestion with 30% hydrogen peroxide (H_2_O_2_ 130 Vol. for 48 h); **F** detail of the fragment surface showing deep cracks; on the right in red (I), the corresponding Raman spectrum; **G**, **H**, **I** in all the collected spectra, no bands shift or significantly broadening effects can be detected, confirming that the adopted procedures have not significantly altered the polymeric structure of the PET fragments

**Fig. 3:**
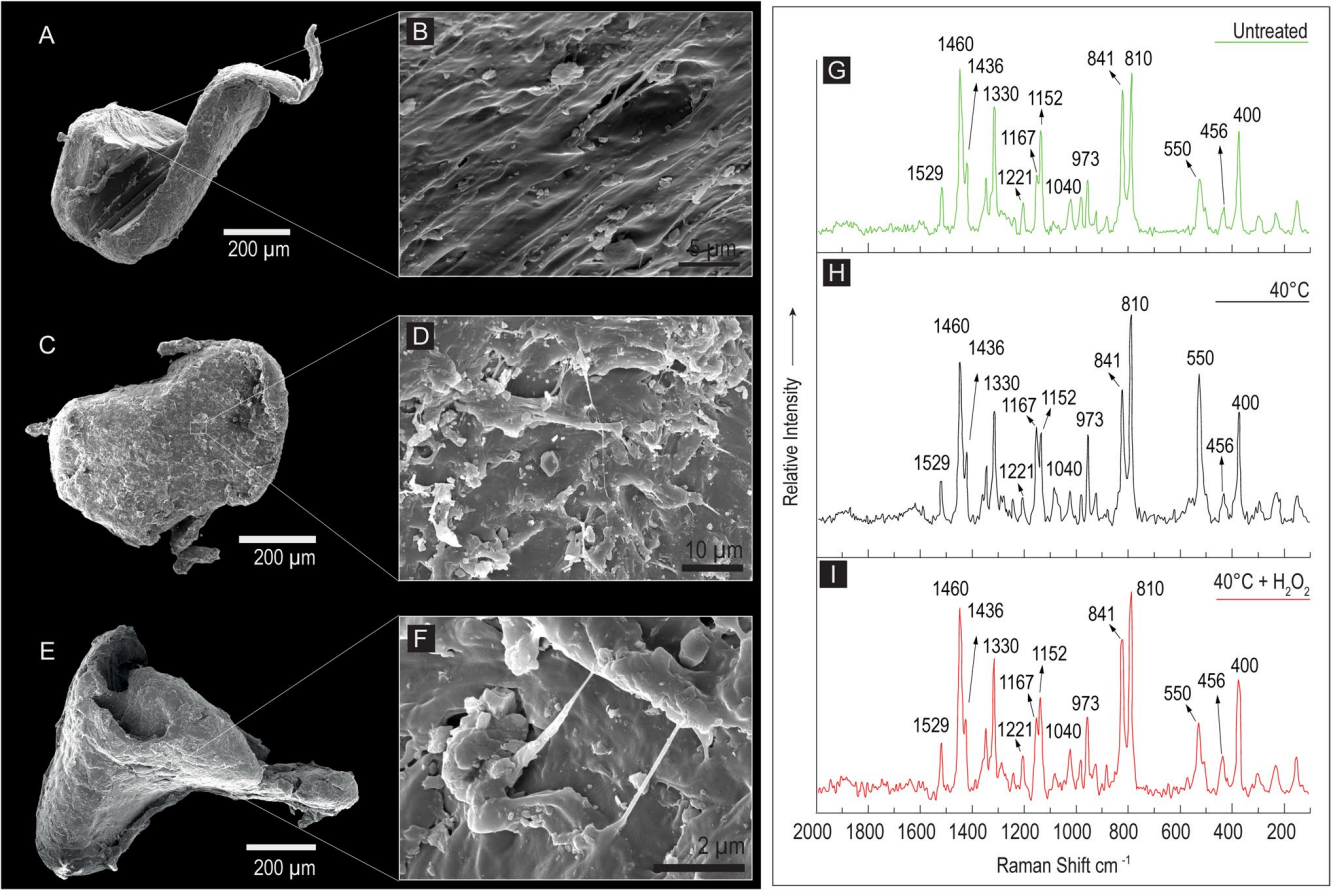
SEM images in secondary electrons and Raman spectra of three polypropylene (PP) fragments. **A** An untreated PP fragment with a drop-like shape; **B** detail of the fragment surface, characterized by a dense linear texture and several sub-micrometer particles; on the right in green (G), the corresponding Raman spectrum. **C** A PP fragment observed after the drying process (48 h at 40 °C); **D** detail of the surface; on the right in black (H), the corresponding Raman spectrum. **E** An irregular shaped PP fragment observed after drying and digestion with 30% hydrogen peroxide (H_2_O_2_ 130 Vol. for 48 h); **F** detail of the fragment surface; on the right in red (I), the corresponding Raman spectrum; **G**, **H**, **I** in all the collected spectra, no bands shift or significantly broadening effects can be detected, confirming that the adopted procedures have not significantly altered the polymeric structure of the PP fragments

**Fig. 4:**
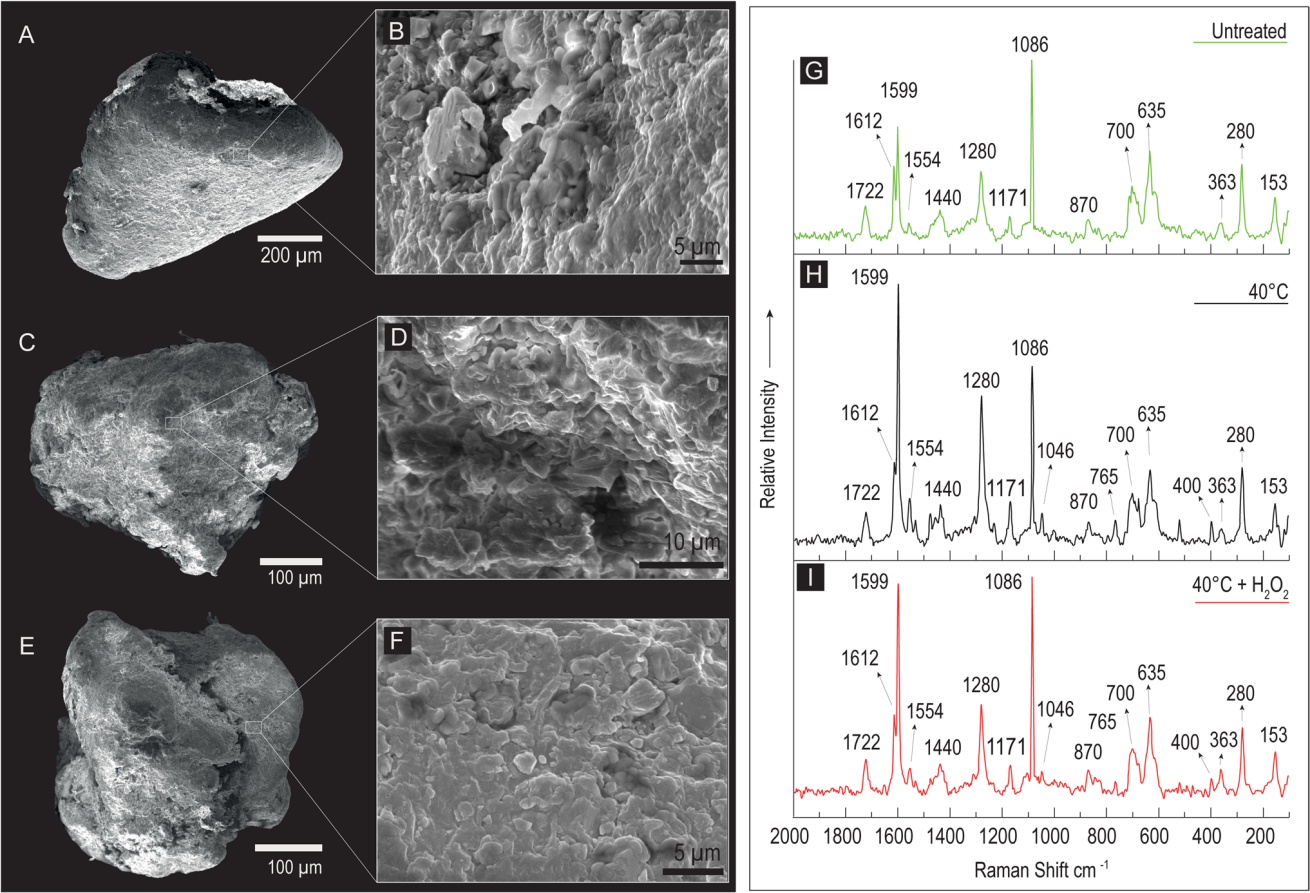
SEM images in secondary electrons and Raman spectra of three Polyvinyl chloride (PVC) fragments. **A** An untreated PVC fragment with a regular shape; **B** detail of the fragment surface, characterized by a granular texture and several sub-micrometer particles; on the right in green (G), the corresponding Raman spectrum. **C** A PVC fragment observed after the drying process (48 h at 40 °C); **D** detail of the surface; on the right in black (H), the corresponding Raman spectrum. **E** An irregular shaped PVC fragment observed after the drying and digestion with 30% hydrogen peroxide (H_2_O_2_ 130 Vol. for 48 h); **F** detail of the fragment surface; on the right in red (I), the corresponding Raman spectrum; **G**, **H**, **I** in all the collected spectra, no bands shift or significantly broadening effects can be detected, confirming that the adopted procedures have not significantly altered the polymeric structure of the PVC fragments

**Fig. 5:**
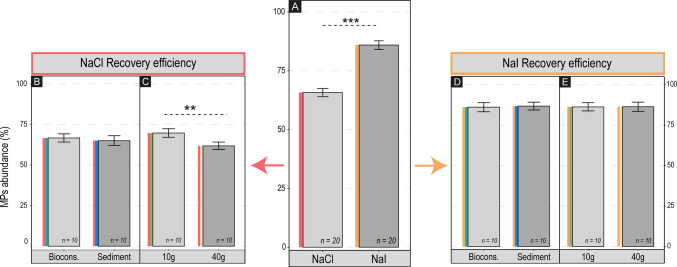
**A** Extraction efficiency as % abundance of recovered MPs, tested by comparing the two salt solutions. **B**, **C** Extraction efficiency for NaCl solution tested on the two substrates and two weights. **D**, **E** Extraction efficiency for NaI solution tested on the two substrates and two weights

**Fig. 6:**
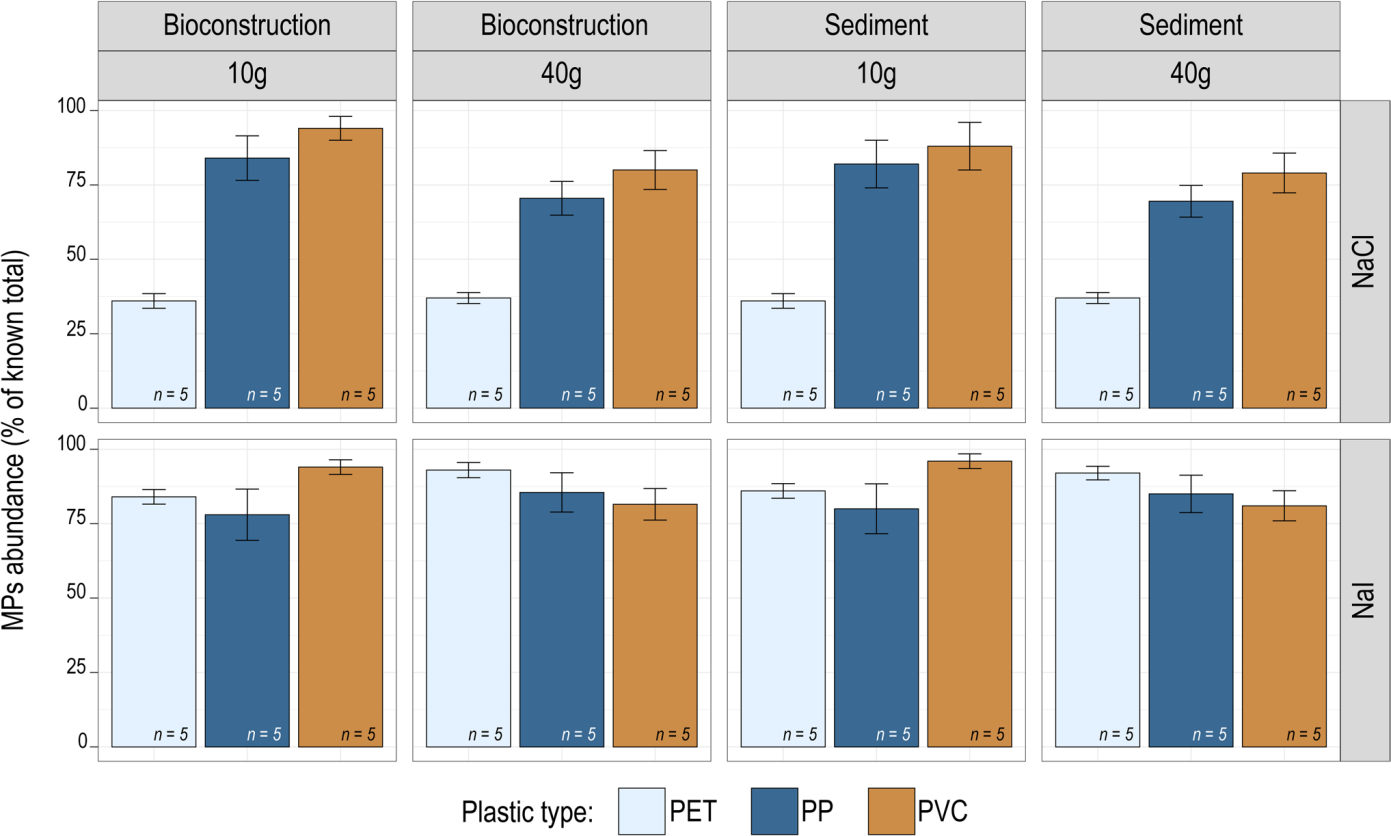
Extraction efficiency of the two salt solutions with respect to the different plastic polymers (PET, PP, and PVC) and across the different substrates and weights (mean ± std. dev.; *n* = 5). Note how the extraction efficiency of NaCl significantly decreases for PET (the densest plastic polymer)

**Fig. 7:**
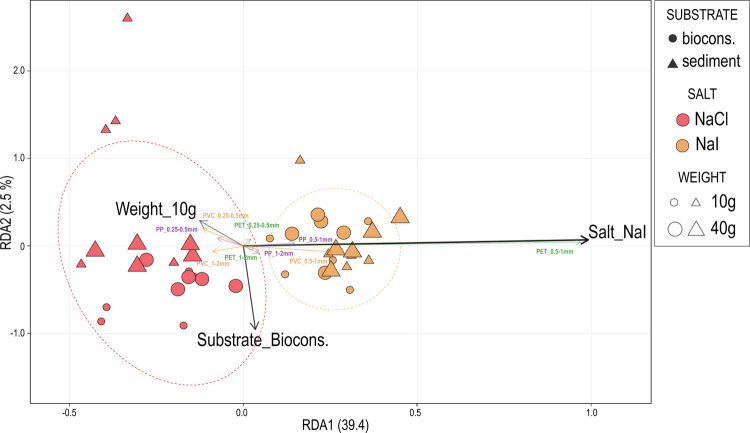
Redundancy analysis (RDA) of MP composition from different extraction protocols. Orange, green, and purple vectors represent different polymer types classified by size fractions. Black vectors indicate experimental factors (substrate, salt, and sample weight). Ellipses show 95% confidence intervals for salt treatments

Secondary electron images of PET, PP, and PVC fragments reported in Figs. 2, 3, and 4A–F reveal the mechanical effects of the grinding/milling procedures. These procedures not only produced near-isodiametric fragments, but also created elongated particles. This was particularly evident in the heterogeneously shaped fragments produced from PET (Fig. 2A, C, E) and PP (Fig. 3A, C, E), which were quite different from the predominantly near-isodiametric fragments of PVC (Fig. 4A, C, E). Fragments of all polymer types at all extraction protocol stages displayed cracks, scratches, and pores on the surface. Moreover, high magnification images clearly show increased surface roughness (Figs. 5, 6, 7B, D, F), where several sub-micrometer particles (nanoplastics) and a fibrous surface (sensu Liu et al. 2016) are shown. No significant morphological alterations were detected after 40 °C treatments and H_2_O_2_ digestions in any of the three polymers.

µ-Raman spectroscopy was applied to the polymers in order to compare the bands’ position and relative intensity after the three extraction steps and to assess possible alterations in polymeric atomic bonds. Fig. S1A–C (available in the supplementary materials) shows the raw spectra of the three untreated polymers, namely orange PVC, light blue PET, and dark blue PP, acquired using two different laser sources: 532 nm and 785 nm. PP bands, both in the fingerprints and CH-stretching regions, are clearly visible in both spectra (Fig. S1B); however, the signal-to-noise ratio was optimized by the 532 nm laser and the spectrum is consistent with the literature for PP Raman features (Anger et al. 2018). On the other hand, in both PET and PVC spectra (Fig. S1A, C), the 532 nm source proved less efficient due to strong fluorescence interference. This effect could not be correlated directly with a different polymeric structure, but is likely related to the interference of the plasticizers and colorants—the characteristic of the plastics used in the experiment. For the same reason, the CH-stretching spectroscopic region proved non-diagnostic with both lasers. Thus, only the 785 nm source proved suitable for the diagnostic analysis of PET and PVC, yielding spectra consistent with the literature (Anger et al. 2018; Nava et al. 2021), while PP was better analyzed using a 532 nm laser source. This preparatory Raman study was crucial for a proper interpretation of potential band shifting/broadening in the step where the different spectra acquired on the same polymer but at different extraction stages were compared. These results highlight a significant challenge for automated µ-Raman analysis of environmental MPs. The necessity of using different optimal laser parameters for different plastic polymers conflicts with the fixed parameters typically required for high-throughput automated systems (Ivleva 2021; Käppler et al. 2016).

Regarding the extraction protocol steps, Figs. 2G, 3G, and 4G show the overlapping spectra of the untreated, 40 °C, and 40 °C + H_2_O_2_ particles in green, red, and black lines, respectively. All three polymers in all three conditions show the same bands with nearly identical relative intensities, not only in the fingerprint range but also in the CH-stretching range, as seen in the PP spectra (Fig. 3G–I).

This result clearly confirms that the preliminary procedures applied did not significantly affect the polymer’s chemical structure, unlike other types of preparation/digestion/treatment (Liu et al. 2022; Prata et al. 2019).

### Recovery efficiency

A total of 2544 MPs were successfully extracted by density separation; MPs had average sizes ranging between 0.625 and 0.675 mm, and more specifically, 0.25–1.75 mm for PET, 0.35–1.625 mm for PP, and 0.25–1.50 mm for PVC. The small differences in the size ranges of the three polymers could be reasonably attributed to the elongated shapes of some MP fragments that may have passed through sieve openings.

Extraction efficiency varied significantly depending on the used salt solution (ANOVA, *F* = 5.617; *p* = 0.003). The NaI solution yielded a significantly higher mean recovery (86.33 ± 8.20%) compared to the NaCl solution (66.08 ± 8.76%) (Fig. 5A) by observing the mean across all polymers, substrates, and weights (*n* = 20 per salt). This result is consistent with previous studies (Quinn et al. 2017). The higher efficiency of NaI was also observed across substrates (bioconstruction *vs* sediment) (Fig. 5B, D) as well as weights (10 g vs 40 g) (Fig. 5C, E).

Nonetheless, a marginally significant difference was observed in samples treated with the NaCl solution (ANOVA, *F* = 4.593, *p* = 0.048). Although this result meets the conventional *p* < 0.05 threshold, its proximity to the significance limit suggests that the observed difference between small (10g: 70.0 ± 8.31%) and large (40g: 62.16 ± 7.19%) samples should be interpreted with caution (Fig. 5C). By contrast, in the case of NaI solution, recovery efficiency is similar for both sample sizes (86.33 ± 7.92% vs 86.3 ± 8.89%; *p* > 0.05) (Fig. 5E).

Although current literature does not clearly refer to sample mass variation as a primary variable in density separation efficiency, the comparatively lower efficiency of the NaCl solution here observed when processing larger samples suggests otherwise. We supposed that the low density of the NaCl solution could facilitate a more rapid sedimentation of heavier grains and cause denser MPs to settle more quickly, leading to their entrapment and burial within the sediment matrix. Potentially, within a larger sediment volume, increased physical entanglement due to greater particle–particle interactions and complex fluid dynamic pathways could develop. Despite the weakly significant difference, these results suggest that matrix overload effects hinder efficient separation in the NaCl brine solution. Consequently, processing larger samples to enhance environmental representativeness may paradoxically compromise recovery rates due to such matrix interference. This finding warrants further investigation into optimized procedures, such as enhanced or prolonged agitation methods (e.g., longer stirring durations or sonication times) to possibly mitigate the observed effects.

Our experiment also shows that the recovery percentage of denser polymers, such as PET (density ~ 1.38 g cm^−3^), was moderate across both substrates and weights when using the NaCl solution (pooled mean *n* = 20: 36.5 ± 4.47%) whereas it was significantly higher with the NaI solution (pooled mean *n* = 20: 85.37 ± 4.28%) (Fig. 6). This clearly demonstrates the limitation of the NaCl solution for the efficient extraction of plastic polymers with densities exceeding ~ 1.20 g cm^−3^. Similar conclusions were reached by other authors (e.g.,Nuelle et al. 2014; Constant et al. 2021) although those studies employed specific extraction devices and focused solely on sediments.

Conversely, the PP fragments (density ~ 0.90 g cm^−3^) being significantly less dense than both saline solutions showed high recovery percentages (NaCl, 76.5 ± 15.33%; NaI, 82.12 ± 15.79%; *n* = 20 each). Similarly, the PVC (density ~ 1.40 g cm^−3^) showed surprisingly high and comparable recovery percentages in both solutions (NaCl, 87.00 ± 14.10%; NaI, 88.13 ± 10.9%; *n* = 20 each). Although the density of PVC is higher than that of the saturated NaCl solution, the recorded high extraction efficiency recorded might be due to other factors, such as plastic fragments morphology (which can influence buoyancy) and surface smoothness (which could prevent the adherence of small sediment particles that would otherwise increase the apparent density of the MP). The high recovery percentages recorded for PP and PVC generally align with the values from the literature (68.80–97.50% (Claessens et al. 2013); 68.0–99.0% (Nuelle et al. 2014)).

When considering the two tested sample weights, extraction efficiencies highlighted a potentially critical role of the salt solution used, particularly for PET fragments (Fig. 6). Recovery using the NaI solution was substantially higher (10 g, 85.0 ± 5.27%; 40 g, 87.50 ± 3.54%) than that obtained using NaCl (10 g, 36.0 ± 5.16%; 40 g, 37.5 ± 3.87%). By contrast, PP and PVC generally showed high recovery percentages with both salts; in 10 g samples, PP had a recovery of 83.0 ± 16.36% (NaCl) and 79.0 ± 17.91% (NaI), while PVC reached 91.2 ± 13.70% (NaCl) and up to 95.0 ± 5.27% (NaI).

When increasing sample weight from 10 to 40 g, recovery percentages decreased slightly for PP (from 83.0% to 70.5 ± 11.60% with NaCl) and PVC (from the ~ 91–95% range to ~ 79.5–80.0% depending on the salt). Notably, these recovery rates were remarkably consistent across both bioconstruction and sediment substrates when other conditions were identical.

Multivariate analyses revealed that experimental factors significantly structured MP recovery patterns (RDA, *F* = 9.07, *p* < 0.001), explaining 43.0% of the total variance. Salt type was the most influential factor accounting for 38.8% of the variance (*F* = 24.49, *p* < 0.001) and clearly separating the samples in the RDA ordination (Fig. 7). PET recovery was strongly associated with the NaI solution, whereas the recovery patterns of PP and PVC were less differentiated.

PERMANOVA confirmed the significant main effect of salt type (Pseudo-*F* = 28.477, *p* < 0.01) and detected significant salt × substrate (*p* = 0.01) and between salt × weight (*p* = 0.014) interactions (Tab. S1, available as supplementary material).

However, PERMDISP tests indicated that these results should be interpreted with caution due to significant heterogeneity in multivariate dispersion, particularly for the NaCl solution (*p* = 0.0168) (Tab. S2, available as supplementary material). Within the NaCl group, recovery patterns showed significantly different variability between substrates (*p* = 0.032) and weights (*p* = 0.046). In contrast, the NaI group was more homogeneous. This suggests that the observed effects are driven by differences in both average recovery (location) and consistency (dispersion), with the NaI solution demonstrating greater methodological robustness.

### The proposed protocol: pros and cons

**Fig. 8:**
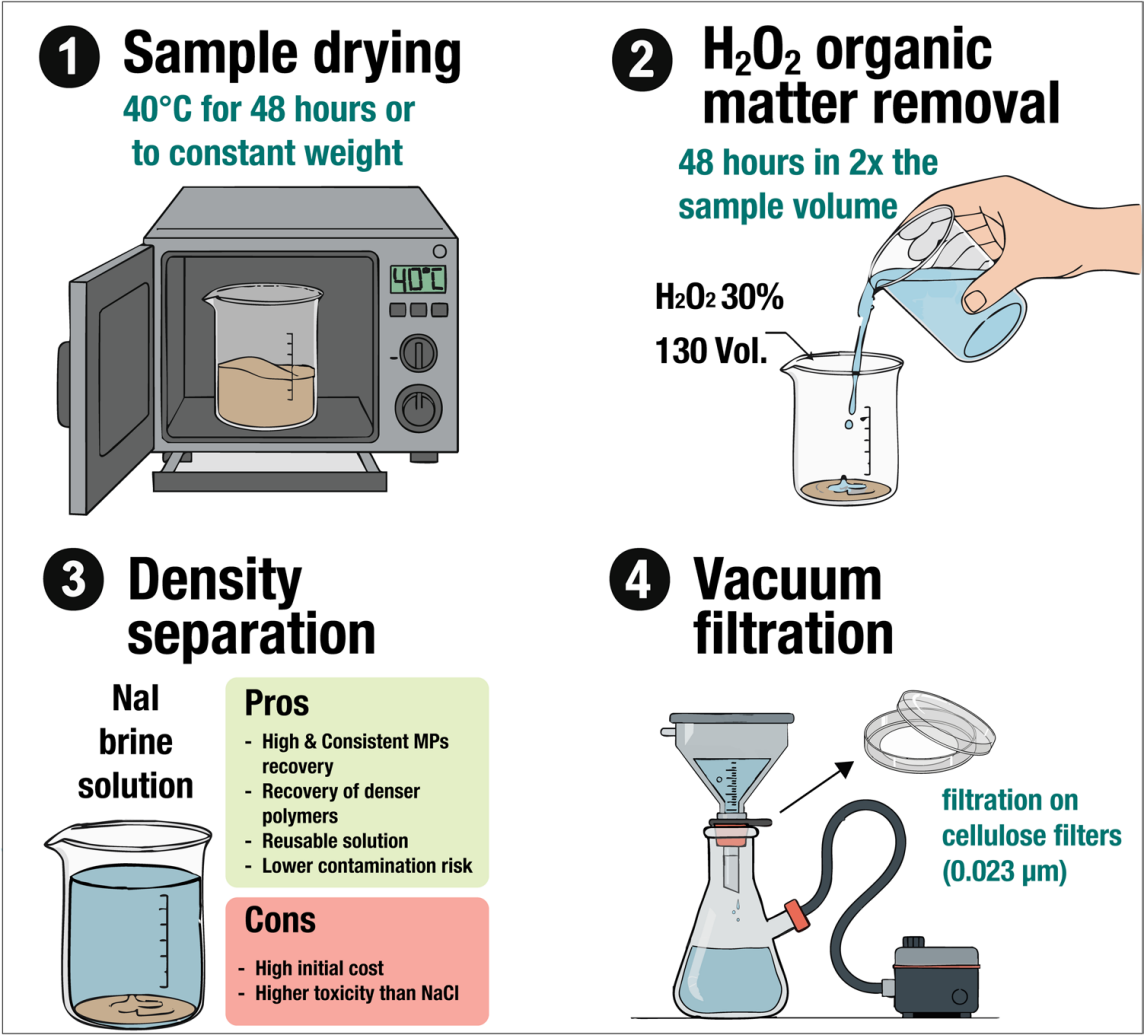
Main procedural steps of the validated protocol

The proposed protocol was specifically designed for biogenic agglutinated matrices; it is synthesized in Fig. 8 and consists of four main steps as follows: (1) sample drying in an oven at 40 °C for at least 48 h, (2) digestion with 30% hydrogen peroxide for about 48 h to totally disaggregate the arenaceous tubes and release MPs, (3) MP density separation using a Sodium Iodide hypersaline solution, and (4) MP extraction by vacuum filtering on cellulose ester Millipore filters with pore size of 0.023 µm.

Our data unequivocally indicates that the sample drying and digestion—essential procedures for studying agglutinated matrices—do not significantly alter MP polymers if conducted under controlled conditions: low temperatures (40 °C), non-aggressive organic matter removal (hydrogen peroxide), and limited application time (48 h). This finding complements the conclusions of Hurley et al. (2018), who noted that H₂O₂ used at elevated temperatures (60–70 °C) could cause significant degradation of sensitive plastic polymers, such as polyamide and polystyrene. The use of more aggressive reagents (e.g., the Fenton’s reagent, which combines H₂O₂ with an iron catalyst, or a multistep Fenton reagent combined with nitric acid) is recommended for digesting samples with a very high organic substance content, for example, in fecal samples (Hurley et al. 2018; Yan et al. 2020). However, data regarding the potential alteration induced to plastic polymers contained in these matrices are lacking. Moreover, these reagents are more expensive than hydrogen peroxide and require specific equipment and particular attention during application, because they are potentially hazardous to human health.

Our result highlights the importance of matrix-specific procedures and confirms that, in the case of bioconstructed agglutinated matrices, an H₂O₂ digestion at low temperatures can ensure the accurate release of unaltered MPs that is essential to obtain reliable abundance data.

The recovery rate obtained with NaI (86.33 ± 8.2%) is comparable with other studies, falling within the broader range of 82.2–100% reported for NaI (Nuelle et al. 2014; Constant et al. 2021; Nava & Leoni 2021). This suggests that the proposed protocol achieves competitive extraction efficiency and offers advantages in terms of simplicity and applicability across different substrate types. The significantly higher performance of NaI highlights the critical importance of selecting appropriate density separation solutions based on target polymer compositions.

Regarding the choice of salt, while sodium chloride (NaCl) was initially suggested by early technical reports (e.g., Galgani et al. 2013) due to its low cost and toxicity, the most recent European guidelines (MSFD Technical Group on Marine Litter, 2023) explicitly acknowledge the limitations of its lower density (~ 1.2 g cm^−3^) for extracting denser polymers. Our findings support this updated perspective: relying solely on NaCl can lead to a substantial underestimation of MP abundance, especially for denser polymers like PET, which is one of the most abundant polymers in the environment. Such underestimation could directly impact the accuracy of risk assessments and the efficacy of policy decisions aimed at managing marine litter. Furthermore, NaCl protocols often require multiple extractions per sample compared to the single extraction possible with NaI. This increases sample handling, processing time, and potential contamination risks. The higher cost of NaI might seem disadvantageous, but this can be offset through solution recovery for reuse in subsequent analyses (Kedzierski et al. 2017), a practice that also mitigates environmental concerns regarding NaI toxicity.

The observation of marginally significant reduced recovery rates in samples with higher weights, particularly for certain polymers and when applying NaCl extraction, cautiously suggests that sample quantity should be carefully evaluated when designing an extraction protocol. Indeed, high quantities of collected samples do not necessarily equate to higher analytical accuracy (particularly in terms of the representativeness of extracted MPs). This result is particularly important for all those “living” substrates protected by specific directives where massive sampling is not ethically sustainable, such as bioengineered reefs.

Finally, in our protocol, we used only common laboratory equipment (beakers, a vacuum filtration column, and a laboratory hood), making it broadly applicable without the need for specialized, high-cost labware.

Nevertheless, our protocol has limitations that highlight the need for further insights. The validation experiment focused only on three specific polymers (PET, PP, PVC) primarily investigated as fragments. Future work should encompass a broader diversity of plastic polymers including fibers and films which may exhibit different extraction dynamics during density separation. Furthermore, the MPs utilized in the spiking experiment were relatively pristine and coarse in size (from 0.250 to 1.41 mm) compared to altered and heterogeneous MPs present in environmental samples.

## Conclusions

Bioengineered agglutinated reefs have been shown to act as accumulation zones for sediments and nutrients in the littoral environment (Gruet 1986; Curd et al. 2019) and, unfortunately, also for MPs (Lo Bue et al. 2023; 2025). Given the multiple ecosystem services associated with these bioengineered reefs, from enhancing biodiversity to providing coastal protection, it is imperative to correctly assess MP pollution to ensure their conservation.

The proposed protocol specifically designed to extract and quantify MP from agglutinated bioconstructed matrices and herein validated through a laboratory experiment represents a further step towards a more accurate evaluation of MP pollution in coastal habitats

## Supplementary Information

Below is the link to the electronic supplementary material.

ESM 1 (DOCX 532 KB)

## Data Availability

The datasets analyzed during the current study are fully available from the corresponding author on reasonable request.
