# Supplementary material for: Microplastic quantification in Sabellaria reefs: a validated protocol for extraction from biogenic agglutinated matrices
Source: Environ Sci Pollut Res Int. 2026 Mar 14;33(11):4939–53. doi: 10.1007/s11356-026-37573-7 (PMC13056732; doi:10.1007/s11356-026-37573-7)
Supplement: Supplementary file 1 — (DOCX 532 KB) [file 11356_2026_37573_MOESM1_ESM.docx]

**Supplementary materials to:**

**Towards reproducibility: sample preparation and microplastic extraction on complex biogenic matrices.** Authored by: Giusto Lo Bue, Rosa Maria Festa, Maya Musa, Maria Pia Riccardi, Alessandro Croce, Agnese Marchini, Nicoletta Mancin


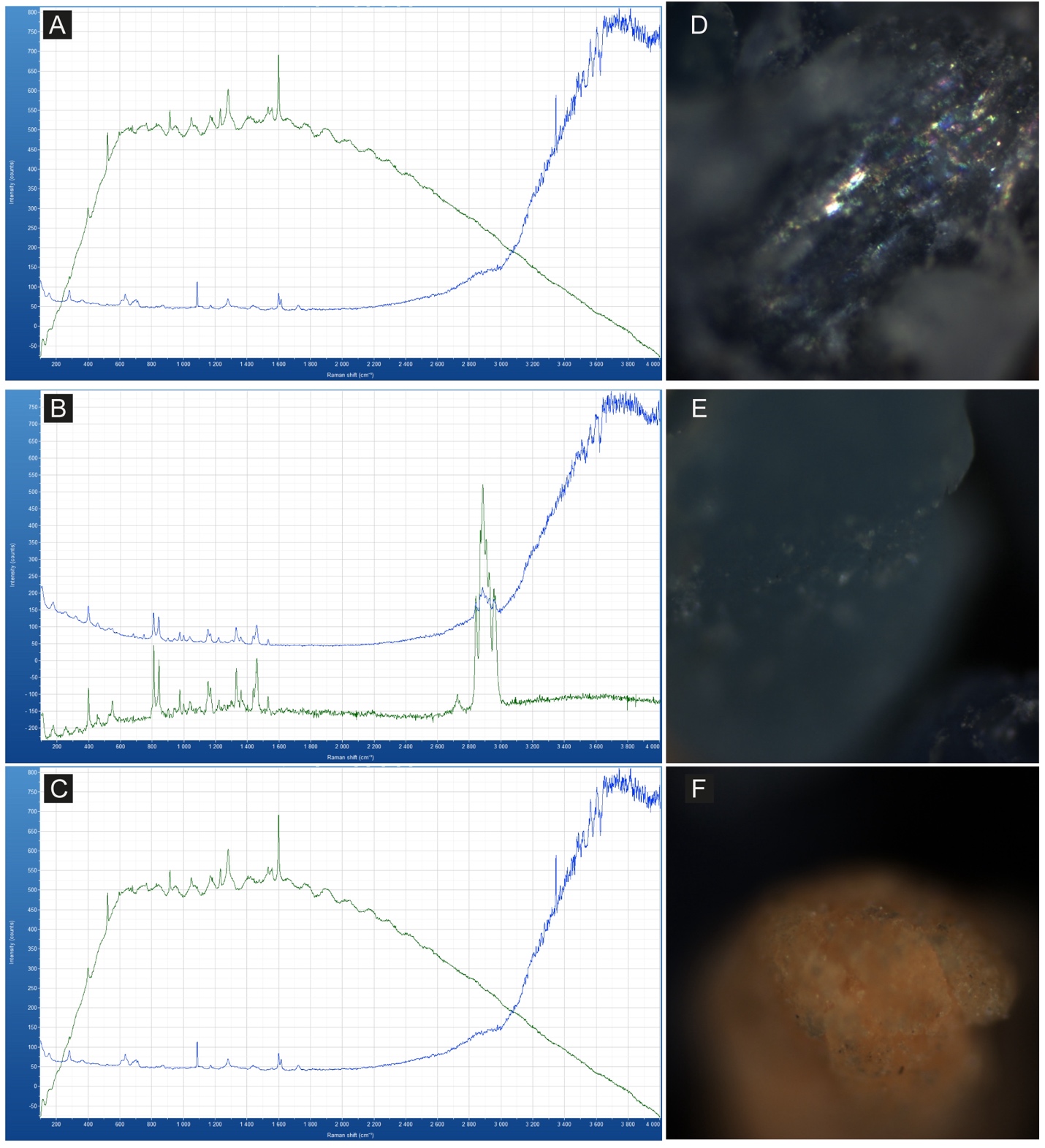


Figure S1: raw spectra of the three untreated polymers acquired by the two different lasers source 532 and 785 nm, in green and blue lines, respectively. A-D) Dark Blue PET; B-E) light Blue PP; C-F) orange PVC.

**Multivariate Analysis results:**

The RDA analysis revealed that the experimental factors (salt types, substrates and sample weights) significantly structured the MPs recovery patterns (Overall model: F=9.07, p<0.001), explaining 43.0% of the total variance. Salt type resulted the most important factor, explaining 38.8% of the variance (F=24.49, p<0.001). The RDA ordination plot (Fig. 4) confirmed this result, showing a clear separation of samples along the primary axis (RDA1, 39.4% variance) based on the used saline solution. PET fragments, particularly the ones falling in the 0.5-1 mm size-class, were strongly associated with the ordination space of the NaI salt, consistent with the univariate results showing higher recovery efficiency of PET with the higher-density salt solution (Fig. 3). Conversely, PP and PVC fragments clustered nearer the ordination centre, indicating that their recovery was less differentiated by salt type or other main factors, aligning with their relatively similar recovery percentages previously observed by univariate analysis (Fig. 3). The PERMANOVA analysis corroborated the significant main effect of salt type (Pseudo-F=28.477, p<0.01) but also detected significant interaction effects between salt and substrate type (Pseudo-F=3.799, p=0.01) and between salt type and sample weight (Pseudo-F=3.799, p=0.014). These interactions clearly indicate that the effect of substrate and weight on recovery patterns was dependent on the used saline solution. PERMDISP tests were conducted to evaluate the homogeneity of multivariate dispersions among the groups defined by the experimental factor. A significant difference in multivariate dispersion among the main factor “Salt” (t= 2.6299, p=1.68E-02) can be observed. Specifically, significant heterogeneity of dispersion was found between substrates (F=4.757, p=0.032) and between weights (F=4.576, p=0.046) in case of NaCl solution. Conversely, the dispersion was slightly heterogeneous both between substrates (F=4.576, p= 0.0485) and between weights in case of NaI solution (F=2.434, p=0.138). Therefore, the significant effects detected by PERMANOVA, particularly the main effect of “Salt” and the interaction terms, must be interpreted with caution as they likely reflect differences in both multivariate location (i.e., average recovery patterns) and dispersion (i.e., variability in recovery patterns consistency). The clear separation observed in the RDA between NaCl and NaI solutions is likely driven by both the different average recovery profiles (especially for PET) and the significantly greater average sample dispersion within the NaCl group (mean deviation 0.357) compared to the more consistent NaI group (mean deviation 0.255). This dispersion heterogeneity provides further context for the significant PERMANOVA interactions and subsequent pairwise tests. The finding that significant differences between substrates (t=1.879, p=0.008) and between sample weights (t=1.655, p=0.0318) occurred only in case of the NaCl solution may be partly attributed to differences in dispersion between these subgroups under the less effective NaCl separation conditions, in addition to shifts in the average recovery profiles. By contrast, the homogeneity of dispersions observed for comparisons within the NaI group supports the interpretation that the non-significant PERMANOVA pairwise tests (for substrate and weight effects within NaI) reflect true similarity mainly in centroid location, further highlighting NaI's methodological robustness under these conditions.

**Table S1:** Main PERMANOVA test results.

| **Source** | **df** | **SS** | **MS** | **Pseudo-F** | **P(perm)** | **Unique perms** | **P(MC)** |
| --- | --- | --- | --- | --- | --- | --- | --- |
| SALT | 1 | 2.7938 | 2.7938 | 28.477 | **0.0001** | 9952 | 0.0001 |
| SUBSTRATE | 1 | 0.17687 | 0.17687 | 1.8028 | 0.119 | 9955 | 0.1311 |
| WEIGHT | 1 | 1.32E-01 | 0.13155 | 1.3409 | 2.39E-01 | 9940 | 0.2386 |
| SALT:SUBSTRATE | 1 | 0.37273 | 0.37273 | 3.7993 | **0.01** | 9924 | 0.0134 |
| SALT:WEIGHT | 1 | 0.35962 | 0.35962 | 3.6657 | **0.0141** | 9940 | 0.0135 |
| SUBSTRATE:WEIGHT | 1 | 0.13963 | 0.13963 | 1.4232 | 0.2134 | 9947 | 0.2146 |
| SALT:SUBSTRATE:WEIGHT | 1 | 0.0946 | 0.0946 | 0.96439 | 0.4107 | 9950 | 0.3988 |

**Table S2:** Pair-wise test Term 'Salt x Weight' for pairs of levels of factor 'Weight' and Term 'Salt x Substrate' for pairs of levels of factor 'Substrate'

| **Within level 'NACL' of factor 'Salt'** | |  |  |  |
| --- | --- | --- | --- | --- |
| **Groups** | **t** | **P(perm)** | **Unique perms** | **P(MC)** |
| 10g, 40g | 1.6556 | **3.18E-02** | 9955 | 0.0451 |
| REEF, SED | 1.8799 | **8.00E-03** | 9950 | 0.0185 |
| **Within level 'NAI' of factor 'Salt'** | |  |  |  |
| **Groups** | **t** | **P(perm)** | **Unique perms** | **P(MC)** |
| 10g, 40g | 1.4337 | 7.33E-02 | 9932 | 0.0887 |
| REEF, SED | 1.1916 | 2.20E-01 | 9936 | 0.223 |
